# Is There a Link Between Older Adults’ Frequency of (Face-to-Face and Remote) Contact With Grandchildren and Cognitive Functioning Over 12 Years?

**DOI:** 10.1093/geronb/gbae175

**Published:** 2024-10-26

**Authors:** Flavia S Chereches, Nicola Ballhausen, Gabriel Olaru, Erika J Laukka, Yvonne Brehmer

**Affiliations:** Department of Developmental Psychology, Tilburg University, Tilburg, The Netherlands; Department of Developmental Psychology, Tilburg University, Tilburg, The Netherlands; Centre for the Interdisciplinary Study of Gerontology and Vulnerability, University of Geneva, Geneva, Switzerland; Department of Developmental Psychology, Tilburg University, Tilburg, The Netherlands; Aging Research Center, Department of Neurobiology, Care Sciences and Society, Karolinska Institutet, Stockholm, Sweden; Stockholm Gerontology Research Center, Stockholm, Sweden; Department of Developmental Psychology, Tilburg University, Tilburg, The Netherlands; Aging Research Center, Department of Neurobiology, Care Sciences and Society, Karolinska Institutet, Stockholm, Sweden; (Psychological Sciences Section)

**Keywords:** Cognition, Face-to-face contact, Grandparenthood, Remote contact

## Abstract

**Objectives:**

Past research has linked more frequent social contacts with better cognition and slower cognitive decline in older adults. An open question is whether face-to-face and remote contact with one’s grandchildren can be beneficial.

**Methods:**

We analyzed data from the Swedish National Study on Aging and Care in Kungsholmen covering a span of 12 years and 2 age cohorts (young–old <78, *N* = 1100; old–old ≥78 years, *N* = 705). We used latent growth curve models to examine whether frequency of face-to-face or remote grandchild contact was associated with cognitive levels and decline and applied (random intercept) cross-lagged models to investigate if these associations were reciprocal.

**Results:**

Face-to-face contact with grandchildren was positively linked to levels of cognition in young–old adults only. We found no associations with cognitive decline. Results of cross-lagged models suggested that grandparents with better cognition had more face-to-face (for young–old adults only) or remote (for old–old adults only) grandchild contact at subsequent waves. However, more grandchild contact was not associated with later cognition.

**Discussion:**

Our findings suggest that grandparents with better cognition engage more with their grandchildren, but that frequency of grandchild contact is not a protective factor against later cognitive decline in older adults.

After the age of 60, general age-related cognitive decline has been observed for several cognitive functions such as episodic memory (Head et al., 2008), reasoning (Singh-Manoux et al., 2012), verbal fluency (Albert et al., 1988), and executive functions (Kennedy & Raz, 2009). However, older adults differ extensively in exhibiting noticeable losses over time (Stine-Morrow et al., 2022). These individual differences in rate and slope of cognitive decline may be attributed to genetic factors as well as environmental influences such as participation in leisure activities or maintenance of social interactions (Bielak & Gow, 2023; Stine-Morrow et al., 2022).

For social interactions specifically, past studies have shown that older adults who engaged in more social interactions showed better cognitive functioning (Kelly et al., 2017) and slower rates of cognitive decline (Zahodne et al., 2019). This positive link might emerge because social interactions afford opportunities to exercise cognitive abilities such as attention, memory, or executive functions (Ybarra et al., 2008), while also offering a means of receiving social support (Kelly et al., 2017).

## Contact With Grandchildren and Cognition

One type of social interaction that has been increasingly addressed in the context of cognition in older age is grandparenthood. Contact with grandchildren is considered to be beneficial for older adults’ well-being (Danielsbacka et al., 2022; Dunifon et al., 2020; Reitzes & Mutran, 2004), but it might also provide cognitive stimulation (Burn & Szoeke, 2015; Danielsbacka et al., 2022). Past research investigated the positive relationship between contact with grandchildren and cognitive functioning, especially by focusing on grandchild care. For example, Sneed and Schulz (2019) report better cognitive functioning and slower cognitive decline in American caregiving grandparents compared with non-caregiving grandparents. Pan and colleagues (2021) found slower decline in episodic memory in Chinese caregiving grandparents compared with non-caregiving grandparents. Most recently, Caputo et al. (2024), using the same sample as Sneed and Schulz (2019), reported that providing grandchild care was linked to better cognitive functioning, especially for more vulnerable groups (i.e., adults with lower income). However, findings are also ambiguous. For instance, Henning and colleagues (2023) did not find an effect of grandchild care on declines in processing speed in a German sample.

These discrepancies could be the result of cultural differences in what grandchild care entails or how it was operationalized. For instance, Henning et al. (2023) utilized a dichotomous variable (care vs no-care), whereas other studies examined various levels of care (e.g., low intensity, medium, high intensity; Caputo et al., 2024). As highlighted by the latter authors, the extent of the positive effects of grandparenting on cognition might depend on the intensity of care, with grandparents providing medium levels of care (2–5 hr per week) showing the least cognitive decline.

So far, research has focused only on grandchild care and did not examine the mere effect of contact with grandchildren on grandparents’ cognition. Thus, an open question is whether regularly engaging with one’s grandchildren, either face-to-face or remotely (e.g., calls), is also beneficial to older adults’ cognitive functioning. The same mechanisms that were put forward to explain how providing grandchild care would affect cognition could explain the link between meeting grandchildren and grandparents’ cognitive functioning: Contact with grandchildren (a) is deemed meaningful and allows for additional opportunities for social interactions and social support (Liao et al., 2021), (b) could involve more physical exercise (e.g., taking a walk with grandchildren, preparing for their visit; Jennings et al., 2021), and (c) could provide more opportunities for intellectual stimulation (e.g., teaching grandchildren, learning from them; Vanhove et al., 2024; Zeng et al., 2021). In addition, care is usually limited to younger grandchildren. Yet, also interactions with adolescent or adult grandchildren might support older adults’ cognition. In fact, as previously reported, older adults value the time spent with their adult grandchildren, enjoy the opportunity to receive and offer support to them (Sheehan & Petrovic, 2008; Wetzel & Hank, 2020; Winefield & Air, 2010), and report decreased mental health if contact with grandchildren is lost (Drew & Silverstein, 2007).

### Type of Contact With Grandchildren: Face-to-Face Versus Remote Contact

Face-to-face contact with grandchildren is not always feasible, due to, for example, grandchildren living further away. In such cases, older adults often resort to alternative means of staying connected, such as phone or video calls (Arpino et al., 2021, 2022; Winefield & Air, 2010). Thus, remote contact can serve as a compensatory measure for the absence of face-to-face interaction (Quadrello et al., 2005). However, remote contact can also complement face-to-face interaction, given that grandparents who frequently meet their grandchildren are also those who maintain regular remote contact with them (Cherlin & Furstenberg, 1986; Quadrello et al., 2005). So far, the effect of remote contact on grandparents’ cognition remains unclear, and thus we aim to investigate the effects of both face-to-face and remote contact on grandparents’ cognition.

## Cognitive Functioning as a Predictor of Frequency of Contact With Grandchildren

Existing studies on grandparenthood and cognition focus mainly on the effect of grandchild care on older adults’ cognition and cognitive decline and overlook the potential influence of cognition on grandchild contact. It is plausible that cognitively fitter older adults are in fact those that meet their grandchildren more often, or that frequency of contact with grandchildren and older adults’ cognition reinforce each other over time (see Lou et al., 2021, for similar findings on social relations). Such reciprocal effects have been discussed in the context of volunteering, with research reporting that older adults with better cognitive functioning engage in more volunteering but still benefit from it (Kail & Carr, 2020). Thus, in this study we also aim to examine whether associations between contact and cognition are directional or reciprocal.

## The Link Between Contact With Grandchildren and Cognition Among Old–Old–Adults

In addition to a potential reciprocal link between contact with grandchildren and cognition, the nature of these effects may differ between young–old and old–old adults (generally those 80 years of age and older). Increased age-related losses in multiple domains of functionality, including cognition, might amplify the role of social relationships for cognitive health in old–old adults, magnifying the positive effect of contact with grandchildren on cognition. For example, frequent meetings with grandchildren might be particularly important later in life, when cognitive functioning is at greater risk of decline.

However, notions of lifespan theory (Baltes & Smith, 2003; Baltes et al., 1998) suggest that biological constraints might be too pervasive to be outweighed by the benefits of social resources in old–old adults. Therefore, the protective effects of social engagement, or meeting with grandchildren, might lose efficiency for old–old adults. We thus might see no effects of grandchild contact on cognition. Yet, even if grandchild contact might not predict later cognition in old–old adults, the reverse link might still hold—better cognitive performance might predict more grandchild contact at subsequent waves. However, as previous research has not made the distinction between young–old and old–old adults in the context of grandparenthood, this examination will be of an exploratory nature.

## Present Study

This study aimed to examine whether face-to-face and remote contact with grandchildren is linked to levels and decline in cognitive functioning in young–old and old–old grandparents. We examined the effects of contact with grandchildren using objective cognitive measures while also investigating the reverse link (from cognition to grandchild contact).

Given some past findings reporting a positive link between grandchild care and cognition (Caputo et al., 2024; Choi et al., 2024; Sneed & Schulz, 2019), we preregistered the following: Greater frequency of contact with one’s grandchildren is positively correlated with level of cognitive functioning (Hypothesis 1); greater frequency of contact with one’s grandchildren (at baseline) is linked to slower rates of cognitive decline across 12 years (Hypothesis 2). We expected reciprocal associations between the frequency of contact with grandchildren and cognitive functioning, in line with past literature showing reciprocal links between volunteering and cognition (Kail & Carr, 2020). We expected these reciprocal links both at the between- and within-person levels. This was to understand which grandparents had better cognitive functioning at later time-points (between-person findings) and if declines in cognition/frequency of contact with grandchildren lead to follow-up changes in contact with grandchildren/cognition (within-person findings). At the between-person level, we preregistered that (a) more contact with grandchildren would predict better cognitive functioning at subsequent time points (Hypothesis 3A) and (b) better cognitive functioning would predict more contact with grandchildren at subsequent time points (Hypothesis 3B). Similarly, at the within-person level (Luo et al., 2021), we assumed that declines in frequency of contact with one’s grandchildren would predict declines in cognitive functioning at subsequent time-points (Hypothesis 4A) and that declines in cognitive functioning would predict declines in frequency of contact with one’s grandchildren at subsequent time-points (Hypothesis 4B).

We explored these associations in young–old and old–old adults. However, due to a lack of previous research investigating grandparents in very old age, we did not formulate specific hypotheses regarding potentially differential patterns of effects in the two groups.

## Method

### Participants

This preregistered study (https://osf.io/pxsu6/) used data from the Swedish National Study on Aging and Care in Kungsholmen (SNAC-K; Lagergren et al., 2004), an ongoing longitudinal study on older adults (60 years and older) living at home or in institutions on the island of Kungsholmen in central Stockholm, Sweden. A stratified sampling method based on age was used, with participants being followed for up to 12 years by interviews, clinical examinations, and neuropsychological tests administered by nurses, physicians, and psychologists. Cognitive assessments took place every 6 years for the 60-, 66-, and 72-year cohorts, but every 3 years for the 78- and 81(+)-year cohorts.

Of the 3,363 participants who participated in the first assessment from 2001 to 2003, we selected those who completed at least one cognitive assessment, were not diagnosed with dementia or other neurological diseases, and were grandparents at the beginning of the study. The remaining 1,805 participants represented five age cohorts (i.e., 60, 66, 72, 78, and 81+ years at the first measurement occasion). Within this manuscript, we refer to the younger participants (i.e., 60, 66, and 72 year cohorts) as young–old adults (*N* = 1100, *M*_*ag*e_ = 65.80, *SD*_*age*_ = 4.85, 56% being female) and to the older participants (i.e., 78 and 81+ year cohorts) as old–old adults (*N* = 705, *M*_*age*_ = 83.22, *SD*_*age*_ = 5.14, 66% being female). We ran the analyses separately for the two age groups.

### Measures

#### Cognitive measures

Trained psychologists administered a cognitive test battery at each measurement occasion, assessing episodic memory, perceptual speed, semantic memory, and semantic fluency: category fluency and letter fluency (Laukka et al., 2020). Each cognitive domain was evaluated using two distinct tests, except for semantic memory (see OSF for a description of how each cognitive domain was assessed). As a previous parallel analysis on this sample suggested that one common factor of cognitive functioning was sufficient to explain individual differences in the cognitive domains (Olaru et al., 2023), we computed one total cognitive ability score for the subsequent analysis. More specifically, standardized *T* scores (with a mean of 50 and a standard deviation of 10) were computed for each measurement occasion based on the mean and standard deviation at the initial measurement occasion within the respective age groups (young–old and old–old, separately). Subsequently, the nine standardized scores were averaged, resulting in a general cognitive ability score.

#### Contact with grandchildren

##### Face-to-face contact

Participants were asked, “How often do you meet the following in person: Friends, neighbors, parents, children, children-in-law, siblings, other relatives, grandchildren.” They reported the frequency of contact for each social member separately, and data referring to contact with grandchildren was used for data analysis (6-point Likert scale, where 1 = daily, more than twice/week, 2 = weekly, more than twice/month, 3 = monthly, more than 6 times/year, 4 = quarterly, more than once/year, 5 = less often, 6 = never). Responses were reverse coded so that higher values indicate more frequent contact.

##### Remote contact

Participants were asked, “How often are you in touch, via telephone, letters, e-mail with the following”—and were presented with the same social contact list as for face-to-face contact. As before, we considered the response reported for grandchildren (6-point Likert scale, same as for face-to-face contact). We reverse-coded the responses such that higher values indicate more frequent contact.

#### Covariates

We included age (number of years), gender (0 = male and 1 = female), education (number of years of education), work status (working vs not working), grip strength, general frequency, number of waves spent living alone, and frequency of meeting with social network as covariates. Note that for the latter, we averaged both face-to-face and remote contact over all social members (see previously), with the exception of grandchildren, children, and children-in-law who might have been the ones facilitating grandchild contact in the first place (e.g., Tan et al., 2010). As these covariates could not only be a confounder but also mediators for the link between grandchild contact and cognition (e.g., grandchild contact increases health, which increases cognitive performance), we also estimated the models with limited covariates (i.e., only age, gender, and education). We *z*-transformed the scores for the covariates from the first assessment point for each age group (i.e., young old and old old), separately. See OSF for a description on how covariates were assessed and justifications for their inclusion.

### Statistical Analyses

All analyses were run in R version 4.1.3 (R Core Team, 2022), with the R-packages psych v2.3.3 (Revelle, 2020) and lavaan v0.6.15 (Rosseel, 2012). We considered a correlation of *r* ≥ 0.10 to be small, *r* ≥ 0.20 to be moderate, and *r* ≥ 0.30 to be large (Gignac & Szodorai, 2016). For the cross-lagged panel models, we considered cross-lagged effects above 0.03 as small, above 0.07 as medium, and above 0.12 as large (Orth et al., 2022). All models were estimated using full information maximum likelihood to account for missing data.

#### Latent growth curve models

To investigate if grandchild contact protects against cognitive decline, we used latent growth curve models. We used the T-standardized test scores as indicators for each measurement occasion and specified the level factor by constraining all loadings and all measurement occasions to 1. The linear slope (i.e., change) factor was specified by constraining the measurement occasion loadings to an increasing order, that is, from λ = 1 at the second to λ = 4 at the fifth measurement occasion. For the young–old group, which was only assessed every 6 years, the slope had a loading of λ = 2 on the third and λ = 4 on the fifth measurement occasion. Indicator intercepts were constrained to 0, and factor means were freely estimated. We regressed the frequency of contact with grandchildren and the control variables on the cognitive ability level and change factors. In a separate supplementary analysis, we also added a quadratic slope for cognition. Grandchild contact did not significantly predict the quadratic slope in old–old adults. We report these results in the OSF supplemental materials.

#### Cross-lagged panel models

We employed cross-lagged panel models (CLPM) and random-intercept cross-lagged panel models (RI-CLPM) to investigate reciprocal associations. CLPM examines whether adults who have more contact with their grandchildren also have better cognition over time. RI-CLPM includes a random-intercept factor to account for stable between-person differences in grandchild contact and cognition (Hamaker et al., 2015), allowing to see if fluctuations in contact within individuals predict subsequent changes in cognition over time and vice versa. It should be noted that the CLPM reflects a mixture of between- and within-person effects, if both are present (Hamaker et al., 2015). However, as recently outlined, one could still make use of the CLPM when one has a predictive research question—for example, learning which individuals are at risk of having lower cognition later on (Hamaker et al., 2020). Running and comparing results of the two models is therefore informative: In the situation in which the results of the CLPM are not replicated within the RI-CLPM (which controls for stable between-person differences with the random intercepts), one might conclude that the observed effects in CLPM are rather driven by between-person differences.

We constrained the autoregressive, cross-lagged effects and the occasion-specific correlations to equality across time to improve the statistical power and robustness of the estimates (Orth et al., 2021). These constraints were also justifiable, as tested by comparing a model with equality constraints across time to a model without (see OSF Table S1). If change from one wave to the other would have changed direction or would have been significant only between certain timepoints, we would have expected the unconstrained models to fit better. Note that the (RI)CLPMs models for old–old adults were run only on 4 waves of data, due to convergence issues (i.e., due to too few assessments at time point 5).

Model fit indices indicated acceptable fit: LGCMs (CFI = 0.948–0.964; RMSEA = 0.061–0.094; SRMR = 0.016–0.034; see OSF Table S2), CLPMs (CFI = 0.976–1.000; RMSEA = 0.035–0.072; SRMR = 0.016–0.068), and RI-CLPMs (CFI = 0.997–1.000; RMSEA = 0.000–0.021; SRMR = 0.007–0.028; see OSF Tables S1–S4).

To estimate the power for the level and change correlations in the LGCMs and cross-lags in the RI-CLPM as part of the preregistration, we simulated data using the lavaan R-package. For both cohorts and both models, power to detect medium associations was generally on average high (>80%, see preregistration).

#### Deviation from preregistration

We preregistered to control for remote contact when examining the effect of face-to-face contact—and vice versa. However, the association between the two forms of contact was much higher than expected (*r* = 0.62 and 0.67, *ps* < 0.001 for young–old and old–old adults, respectively). We thus decided to investigate the effects of the two separately, without controlling for the other.

## Results

### Descriptive Results

An overview of descriptive statistics and correlations for grandchild contact (face-to-face and remote) and cognition (both overall and each cognitive domain) is presented in Tables 1 and 2 and the OSF. The face-to-face and remote contact retest correlations across 12 years were mostly moderate, on average 0.47 for young–old and 0.51 for old–old adults (all *ps* < 0.01). The cognition retest correlations were on average 0.81 for young–old adults and 0.76 for old–old adults (all *ps* < 0.001). The correlations between grandchild contact and cognition were small in size, and for the old–old adults, the associations disappeared at later assessment waves.

**Table 1.** Means, Standard Deviations and Correlations in Young–Old Grandparents

Variables	*N*	*M*	*SD*	Cog.1	Cog.3	Cog.5	G.1	G.3	G.5	G.R.1	G.R.3	G.R.5
Cog.1	1,100	49.97	6.40									
Cog.3	901	49.60	6.90	0.86^***^								
Cog.5	708	47.50	7.10	0.77^***^	0.81^***^							
G.1	892	4.52	1.04	0.17^***^	0.13^***^	0.16^**^						
G.3	809	4.55	1.00	0.19^***^	0.19^***^	0.19^***^	0.58^***^					
G.5	666	4.52	1.01	0.14^***^	0.13^***^	0.17^***^	0.47^***^	0.56^***^				
G.R.1	812	4.73	1.21	0.10^**^	0.03	0.03	0.62^***^	0.38^***^	0.31^***^			
G.R.3	771	4.65	1.17	0.10^**^	0.07	0.03	0.46^***^	0.56^***^	0.36^***^	0.56^***^		
G.R.5	639	4.45	1.24	0.09^*^	0.06	0.05	0.40^***^	0.44^***^	0.58^***^	0.39^***^	0.43^***^	
Grip strength	1,086	290.74	116.20	0.03	0.06	0.02	−0.08^*^	−0.03	−0.01	−0.19^***^	−0.11^**^	−0.08^*^
Social network	1,100	27.40	9.97	0.17^***^	0.16^***^	0.16^***^	0.15^***^	0.10^**^	0.13^***^	0.22^***^	0.11^**^	0.11^**^
Living alone	1,100	1.10	1.35	−0.05	−0.12^***^	−0.17^***^	−0.03	−0.01	−0.06	0.03	0.05	−0.01
Work (yes)	1,065	0.40	–	0.25^***^	0.25^***^	0.32^***^	0.03	0.10^**^	0.14^***^	0.00	0.02	0.01
**Demographics**												
Gender (w)	1,100	0.56	–	0.10^***^	0.07^*^	0.10^**^	0.17^***^	0.13^***^	0.05^***^	0.25^***^	0.17^***^	0.10^**^
Age	1,100	65.8	4.85	−0.25^***^	−0.32^***^	−0.35^***^	−0.09^**^	−0.09^*^	−0.14^***^	−0.04	0.00	0.00
Education	1,100	13.17	4.02	0.46^***^	0.45^***^	0.36^***^	0.10^**^	0.12^***^	0.10^*^	0.01	0.05	0.04

*Notes*: Cog.1 = Cognition at wave 1; Cog.3 = Cognition at wave 3; Cog.5 = Cognition at wave 5; G.1 = Face-to-face grandchild contact wave 1; G.3 = Face-to-face grandchild contact wave 3; G.5 = Face-to-face grandchild contact wave 5; G.R.1 = Remote grandchild contact wave 1; G.R.3 = Remote grandchild contact wave 3; G.R.5 = Remote grandchild contact wave 5; Living alone = Number of waves spent living alone; *SD* = standard deviation; Social network = Frequency of contact with members of social network, other than grandchildren; Work (yes) where 0 = not working and 1 = working; Gender (w), where 0 = men and 1 = women; Education = Years of education.

^*^ *p* < .05.

^**^ *p* < .01.

^***^ *p* < .001.

**Table 2.** Means, Standard Deviations and Correlations in Old–Old Grandparents

Variables	*N*	*M* *(SD)*	Cog.1	Cog.2	Cog.3	Cog.4	Cog.5	G.1	G.2	G.3	G.4	G.5	G.R.1	G.R.2	G.R.3	G.R.4	G.R.5
Cog.1	705	49.77 (7.02)															
Cog.2	476	49.48 (7.37)	0.84^***^														
Cog.3	341	48.25 (7.84)	0.76^***^	0.87^***^													
Cog.4	229	47.06 (7.73)	0.71^***^	0.77^***^	0.84^***^												
Cog 5	116	46.22 (7.73)	0.63^***^	0.67^***^	0.72^***^	0.83^***^											
G.1	653	4.28 (1.09)	0.11^**^	0.10^*^	0.08	0.09	0.00										
G.2	383	4.27 (1.06)	0.17^**^	0.11^*^	0.21^***^	0.18^*^	−0.05	0.61^***^									
G.3	299	4.14 (1.06)	0.19^**^	0.20^**^	0.15^*^	0.11	−0.04	0.60^***^	0.66^***^								
G.4	193	4.06 (1.09)	0.07	0.05	0.10	0.09	−0.09	0.52^***^	0.57^***^	0.68^***^							
G.5	93	4.13 (1.13)	0.06	0.05	−0.07	−0.08	−0.18	0.50^***^	0.45^***^	0.61^***^	0.61^***^						
G.R.1	623	4.61 (1.17)	0.16^***^	0.13^**^	0.06	0.02	−0.16	0.67^***^	0.45^***^	0.52^***^	0.42^***^	0.44^***^					
G.R.2	373	4.51 (1.18)	0.17^**^	0.16^**^	0.18^**^	0.14	0.02	0.52^***^	0.70^***^	0.61^***^	0.47^***^	0.29^*^	0.53^***^				
G.R.3	280	4.42 (1.24)	0.14^*^	0.14^*^	0.10	0.06	−0.19	0.47^***^	0.52^***^	0.67^***^	0.53^***^	0.49^***^	0.60^***^	0.62^***^			
G.R.4	191	4.17 (1.20)	0.01	0.10	0.08	0.07	−0.06	0.42^***^	0.42^***^	0.48^***^	0.68^***^	0.47^***^	0.44^***^	0.46^***^	0.57^***^		
G.R.5	88	4.11 (1.27)	0.11	0.14	0.04	0.17	0.00	0.42^***^	0.36^**^	0.42^***^	0.36^**^	0.66^***^	0.49^***^	0.37^**^	0.52^***^	0.50^***^	
Grip strength	542	204.92 (87.26)	0.13^**^	0.09	0.20^***^	0.12	−0.08	0.03	−0.06	−0.05	0.04	0.12	−0.05	−0.04	−0.04	0.02	0.01
Social network	705	21.05 (9.78)	0.27^***^	0.24^***^	0.22^***^	0.13^*^	0.13	0.10^**^	0.10^*^	0.08	−0.04	0.22^*^	0.23^***^	0.14^**^	0.17^**^	0.01	0.33^**^
Living alone	705	1.98 (1.65)	0.12^**^	0.12^**^	0.03	−0.04	0.04	0.02	0.02	0.01	−0.10	−0.02	0.09^*^	0.04	−0.07	0.01	−0.05
Work (yes)	701	0.01	0.09^*^	0.08	0.10	0.08	0.04	0.05	0.01	0.00	−0.01	−0.02	0.03	0.05	0.00	0.07	−0.01
**Demographics**																	
Gender (w)	705	0.66	−0.02	−0.01	−0.03	−0.02	0.08	0.07	0.16	0.07	−0.04	0.03	0.11^**^	0.15^**^	0.04	0.07	0.10
Age	705	83.22 (5.14)	−0.36^***^	−0.31^***^	−0.27^***^	−0.29^***^	−0.21^**^	−0.12^**^	−0.14^**^	−0.12^*^	−0.08	−0.13	−0.16^***^	−0.13^*^	0.04	0.00	−0.08
Education	705	10.64 (3.84)	0.43^***^	0.39^***^	0.42^***^	0.34^***^	0.20^*^	0.09^*^	0.09	0.21^***^	0.19^**^	0.17	0.06	0.10	0.18^**^	−0.04	0.13

*Notes*: Cog.1 = Cognition at wave 1; Cog.2 = Cognition at wave 2; Cog.3 = Cognition at wave 3; Cog.4 = Cognition at wave 4; Cog.5 = Cognition at wave 5; G.1 = Face-to-face grandchild contact wave 1; G.2 = Face-to-face grandchild contact wave 2; G.3 = Face-to-face grandchild contact wave 3; G.4 = Face-to-face grandchild contact wave 4; G.5 = Face-to-face grandchild contact wave 5; G.R.1 = Remote grandchild contact wave 1; G.R.2 = Remote grandchild contact wave 2; G.R.3 = Remote grandchild contact wave 3; G.R.4 = Remote grandchild contact wave 4; G.R.5 = Remote grandchild contact wave 5; Living alone = Number of waves spent living alone; *SD* = standard deviation; Social network = Frequency of contact with members of social network, other than grandchildren; Work (yes) where 0 = not working and 1 = working; Gender (w), where 0 = men and 1 = women; Education = Years of education.

^*^ *p* < .05.

^**^ *p* < .01.

^***^ *p* < .001.

Over the 12 years of the study period, univariate LGM models (including only cognition) revealed that cognitive abilities levels decreased on average by 3.44 (young–old) and 9.76 (old–old) *T* scores (both *ps* < 0.001). Face-to-face contact decreased on average by 1.16 (young–old, *p* = .002) and 3.95 (old–old, *p* < .001) *T* scores, and remote contact by 2.61 (young–old) and 5.83 (old–old, all *p* < .001) *T* scores. There were significant individual differences in cognitive change, as indicated by significant variance of the slope parameters (young–old: *SD* = 3.87; old–old: *SD* = 5.66; both *p* < .001), face-to-face contact (young–old: *SD* = 6.21, *p* = .006; old–old: *SD* = 7.64, *p* = .002), and remote contact only in young–old (*SD* = 7.12, *p* = .003). Note that declines based on the score means presented in Tables 1 and 2 are smaller than the ones estimated by the LGM models. This is because participants with lower cognitive levels and stronger decline are more likely to drop out (see OSF for attrition analysis), and later mean scores are based on cognitively fitter participants, who still participated. In contrast, the estimates from the LGM models take into account all participants, including those who may have dropped out over time (i.e., participants with lower cognitive functioning).

### Level and Change Associations

**Figure 1.**
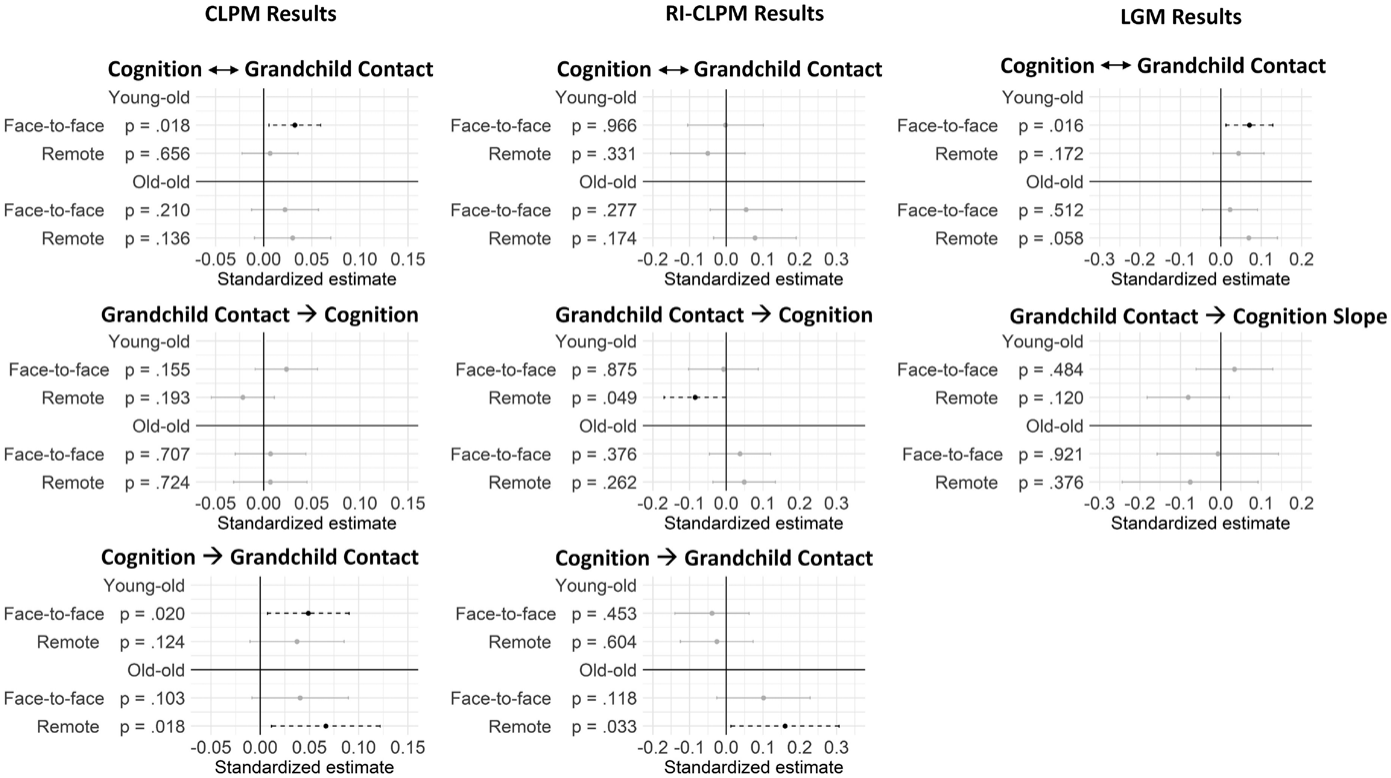
Model results. *Notes*: Face-to-face = face-to-face contact with grandchildren; Remote = remote contact with grandchildren; CLPM = Cross-lagged panel model; (RI)CLPM = Random intercept cross-lagged panel model; LGM = latent growth curve model; CLPM Results: Cognition ↔ Grandchild Contact reflects the within-time points correlations between grandchild contact and cognition; CLPM Results: Grandchild Contact → Cognition reflects grandchild contact predicting cognition at subsequent waves; CLPM Results: Cognition → Grandchild Contact reflects cognition predicting frequency of grandchild contact at subsequent waves; RI-CLPM Results: Cognition ↔ Grandchild Contact reflects residual correlations within each assessment wave between cognition and grandchild contact; (RI)CLPM Results: Cognition → Grandchild Contact reflects cognition predicting frequency of grandchild contact at subsequent waves; (RI)CLPM Results: Grandchild Contact → Cognition reflects grandchild contact predicting cognition at subsequent waves; LGM Results: Cognition ↔ Grandchild Contact reflects associations between levels of cognition and levels of grandchild contact; LGM Results: Grandchild Contact → Cognition Slope reflects grandchild contact predicting change in cognition over time.

First, we examined whether grandchild contact and cognition levels (i.e., intercepts) are associated with each other and if grandchild contact predicted cognitive change (i.e., slope). Associations controlling for the covariates are presented in Figure 1 (see OSF Figure S1 for results with a limited number of covariates). In line with our first hypothesis, we found that face-to-face grandchild contact was (very weakly) associated with cognition levels in young–old adults (ρ = 0.07; *p* = .016), indicating that young–old adults who see their grandchildren more often in person also have better cognitive functioning. However, although we found significant interindividual differences in cognitive changes in both young–old and old–old adults (all *p*s < 0.001), the frequency of in-person and remote grandchild contact at baseline was not associated with individual differences in cognitive decline. Thus, our second hypothesis was not supported. Moreover, most of the covariates included as predictors of cognitive decline were also not significant. An exception is age in both young–old and old–old adults (β = −0.25; *p* < .001, respectively β = −0.27; *p* = .002), and the number of waves spent living alone in young–old adults (β = −0.12; *p* = .004). See OSF Tables S5 and S6 for detailed results.

### Cross-Lagged Associations

We next examined the effects of grandchild contact on later cognition and vice versa using the (random-intercept) cross-lagged panel models. The standardized cross-lagged estimates are presented in Figure 1 (for results with limited covariates, see OSF Figure S1). In the CLPM (i.e., a combination of between- and within-person effects), cognition predicted face-to-face contact only in young–old grandparents (β = 0.05; *p* = .020) at subsequent waves, suggesting that those younger grandparents who had better cognition at one wave were also the grandparents who had more contact with their grandchildren at subsequent waves. Among old–old grandparents, better cognition was linked to more remote contact at subsequent waves (β = 0.07; *p* = .018). Grandchild contact did not predict cognition in either of the two age groups. Similar effects were observed also in the models including limited controls (i.e., age, gender, education). We thus found only partial support for our third hypothesis suggesting a between-person effect from cognition to contact, but no effect from contact to cognition.

Within the RI-CLPM, used to test our fourth hypothesis, we found an effect from cognition to subsequent remote contact in old–old adults (β = 0.16; *p* = .033), suggesting that when old–old grandparents decline in their cognitive performance, they decrease in the extent to which they have remote contact with their grandchildren at subsequent waves. This effect was not present in the models including only age, gender, and education as controls; thus, the effect should be interpreted with caution. No effect was observed from grandchild contact to cognition in the old–old group.

In the young–old group, within-person changes in cognition did not lead to changes in grandchild contact at subsequent wave. However, as seen in Figure 1, remote contact appeared to negatively predict cognition at subsequent timepoints in young–old adults (β = −0.09; *p* = .049). However, this effect was also not present in models including limited controls, and it would also disappear if one takes a more stringent alpha level than 0.05. Thus, we found only limited support for our fourth hypothesis.

## Discussion

In this study, we examined the associations between frequency of grandchild contact and grandparents’ cognitive ability levels and declines over 12 years, as well as reciprocal associations between the two constructs. We found no evidence that contact with grandchildren buffers against cognitive decline. Instead, we observed that young–old grandparents with higher cognitive abilities have more contact with their grandchildren (levels-levels association). From cross-lagged panel models we found one direction of the effects: better cognition was associated with more grandchild contact later on, but not vice versa.

As previously reported with this dataset (Olaru et al., 2023), we found cognitive decline in both groups of older adults over the study period. We also found a decline in the frequency of face-to-face and remote contact in both groups of young–old and old–old grandparents. These findings are in line with past research on face-to-face contact in grandparenthood (Silverstein, & Long, 1998).

### Associations Between Levels of Grandchild Contact and Levels of Cognition

When testing the level–level associations between grandchild contact and cognition, we found that frequency of face-to-face contact with grandchildren was linked to levels of cognition only among the younger cohorts (i.e., 60 to 72 years at the beginning of the study). Previous research has confirmed this link (Arpino & Bordone, 2014; Burn et al., 2014), but limited research has focused on the oldest-old. An exception is the study of Liao et al. (2021), who reported a stronger link between grandchild care and cognition in older compared with middle-aged Chinese grandparents. It is possible that we did not observe an effect among the old–old adults due to the prevalent health and cognitive decline characterizing this age group, which may overshadow any potential benefits from contact with grandchildren. In fact, the descriptive results revealed correlations between cognition and contact during the initial assessment waves in the old–old group, but these associations disappeared at subsequent waves.

### Associations Between Grandchild Contact and Cognitive Decline

We found no link between baseline face-to-face or remote contact frequency and the rate of cognitive decline in either sample, suggesting that contact with grandchildren might not protect against cognitive decline. Our findings echo Henning and colleagues’ (2023) research, who found no effect of grandchild care on cognitive decline in processing speed over nine years. The authors also observed no protective effects from other prosocial activities (i.e., informal caregiving, volunteering) on declines in processing speed and concluded that past expectations of significant cognitive health benefits from prosocial involvement may be overstated. In our study, when looking at the effects of the covariates on cognitive decline (cognition slope), we also did not find a protective effect of general frequency of social interactions on cognitive decline.

Interestingly, however, Henning et al. (2023) and our findings diverge from the findings of Caputo et al. (2024) and Sneed and Schulz (2019), who report that caregiving grandparents have better cognitive performance over time. However, the findings of the latter two papers might be sample specific—as they both use the same dataset (Health and Retirement Study). In addition, cultural and geographical aspects might also be important—for example, the frequency of contact with grandchildren might be dependent on how far away these grandchildren live or the norms characterizing that area, country (Danielsbacka et al., 2022).

It is also possible that the effects are specific only to grandchild care, and frequency of grandchild contact is not enough to protect against cognitive decline. Yet, an aspect one should consider with caregiving is that it reflects a more transitory activity (care takes place only when grandchildren are young), and it might protect against cognitive decline while people perform it (which would be in line with the “use it or lose it hypothesis”; Bielak & Gow, 2023; see similar discussion for volunteering in Henning et al., 2023). Thus, as suggested also by Henning et al. (2023), future studies investigating grandchild caregiving could also aim to look over shorter time-periods (e.g., 1 year). In addition, we assume that the motivation, quality, and context for grandchild contact should also be considered, with the effects taking place only when grandparents want to have contact (see similar discussion for volunteering and well-being; Bjälkebring et al., 2020), when the relationship quality is good, or when also the grandchild wishes to have contact with the grandparents (Hank et al., 2018). Alternatively, it’s possible that the frequency of grandchild contact observed in our study—which lays between a score of 4 and 5 (meaning between “Monthly, more than 6 times/year” and “Weekly, more than twice/month,” see Tables 1 and 2)—may not be sufficient to protect against cognitive decline. More frequent interaction or contact combined with other forms of social involvement might yield more positive effects.

It should be noted, however, that although we found individual differences in cognitive decline, most of our covariates did not significantly predict cognitive decline over the study period either—with the exception of age and number of years spent living alone. This finding aligns with past research highlighting the difficulty of accounting for interindividual differences in cognitive decline (Lövdén et al., 2020).

### Does Contact With Grandchildren Predict or is Predicted by Cognitive Functioning?

Drawing from related literature (e.g., volunteering; Kail & Carr, 2020), we anticipated reciprocal links between frequency of grandchild contact and cognition. However, our analyses using CLPM revealed unidirectional effects, suggesting that older adults with better cognition tend to engage more with their grandchildren. Notably, in young–old grandparents, cognition predicted only face-to-face contact, whereas in old–old grandparents, it predicted only remote contact. The presence of a face-to-face effect solely in young–old grandparents may stem from fewer health-related barriers to meeting grandchildren compared with old–old grandparents and possibly the larger involvement of young–old grandparents in grandchild care or with younger grandchildren (Glaser et al., 2013). Among the old–old grandparents, grandchild care might no longer be needed (note, however we do not know the age of the grandchildren), and those grandparents that have good cognitive functioning might be those that keep and initiate contact with their grandchildren remotely.

When looking at the reciprocal effects within-person, using RI-CLPM models, we no longer observed an effect from cognition to later face-to-face contact in young–old adults, as observed in the classical CLPM model. Thus, this effect was most likely driven by between-person differences. We do find two within-person effects: a positive effect from cognition to remote contact in the old–old group and a negative effect from remote contact to later cognition among the young–old grandparents. Note, however, that both associations were significant only when controlling for all covariates—that is, besides age, gender, and education, also factors such as health, general social involvement, or living situation (i.e., number of waves spent living alone). However, some of these factors might be collider variables, or a variable that is casually influenced by both frequency of contact and cognition, and by controlling for such a variable we might allow for a spurious association to arise (grandchild contact → social involvement/health ← cognition; Rohrer, 2018). For example, we control for frequency of interactions with social network—but as previously reported, grandchild contact and care might predict social involvement (Quirke et al., 2019), and cognition is also known to predict social involvement (Luo et al., 2021). A similar reasoning could also be applied to health. In fact, within grandparenthood literature, the variables chosen as covariates are often not consistent from one study to another, and some of them could also be perceived as a collider or as the mechanism through which the effect on cognition come about (for example, health; Jennings et al., 2021). Thus, in line with past recommendations (Mroczek et al., 2022), we run models with both all and also a minimum number of control variables. We report both results, but more research is needed to replicate our findings from the RI-CLPM models before reliably interpreting them.

### Strengths and Limitations

To our knowledge, this is the first study to investigate the reciprocal associations between frequency of grandchild contact and cognition over multiple years, additionally taking the grandparents’ age and type of contact into account, instead of focusing on grandchild care. The high observed correlation between face-to-face and remote contact might suggest that remote contact is not necessarily a substitute for face-to-face contact but more of a supplement instead (if it was compensatory, a negative association might have been expected). This is in line with some older findings (Cherlin & Furstenberg, 1986; Quadrello et al., 2005) but contradicting other research suggesting that older adults might use alternative approaches to keep connecting with their grandchildren when face-to-face contact is not possible (Arpino et al., 2021, 2022; Winefield & Air, 2010).

Our study has limitations worth noting. Firstly, we lack information on the specific activities grandparents engage in during contact with their grandchildren, a common gap in grandparenthood research (Hank et al., 2018). It is possible that only certain activities performed with grandchildren are linked to cognitive functioning, or that the effect occurs only for grandchild care. We lack information on who initiates the contact (grandparents, parents, or grandchildren), the age of the grandchild, how close grandchildren live to their grandparents, or the relationship quality with them. Future studies should aim to explore these nuances further to better understand how the quality and dynamics of grandparent-grandchild interactions influence cognition in older adults. Studies should also aim to continue studying these associations among old–old grandparents, using larger samples to detect any potential small effects.

Lastly, our study uses a sample from an urban area that is relatively healthy (Stockholm, Sweden). This might affect the generalizability of our findings and the presence of several other protective factors in this sample (e.g., high levels of education, occupation, engagement in various cognitively stimulating leisure activities; see Olaru et al. 2023). In fact, more research is needed to look also at less economically advantaged populations, as grandchild contact and grandchild caregiving might represent the norm, being a readily available form of social interaction (for example, see Jennings et al., 2021).

## Conclusion

In this paper we examined the baseline, longitudinal, and reciprocal associations between grandchild contact (face-to-face and remote) and cognition in young–old and old–old adults. We found a positive association between the level of face-to-face grandchild contact and cognition in young–old adults, suggesting that those grandparents who meet their grandchildren face-to-face more often also have better cognitive functioning. However, we did not find any effects of grandchild contact on subsequent cognitive decline. In cross-lagged models we also found that better cognition predicted more face-to-face contact (for young–old) and more remote contact (for old–old) at subsequent timepoints, and not vice versa.

## Supplementary Material

gbae175_suppl_Supplementary_File_1

## Data Availability

The preregistration, analysis scripts and supplementary materials can be accessed in an OSF https://osf.io/pxsu6/. Data is available upon request and research proposal from https://www.snac-k.se/.
