# Supplementary material for: Is There a Link Between Older Adults’ Frequency of (Face-to-Face and Remote) Contact With Grandchildren and Cognitive Functioning Over 12 Years?
Source: J Gerontol B Psychol Sci Soc Sci. 2024 Oct 26;80(3):gbae175. doi: 10.1093/geronb/gbae175 (PMC11831800; doi:10.1093/geronb/gbae175)
Supplement: gbae175_suppl_Supplementary_File_1 [file gbae175_suppl_supplementary_file_1.docx]

**OSF Supplementary material**

***Is There a Link Between Older Adults’ Frequency of (Face-to-Face and Remote) Contact with Grandchildren and Cognitive Functioning over 12 Years?***

**Description of cognitive assessment**

Trained psychologists administered a cognitive test battery at each measurement occasion, assessing episodic memory, perceptual speed, semantic memory and semantic fluency (Laukka et al., 2020). Each cognitive domain was evaluated using two distinct tests, with the exception of semantic memory.

**Episodic Memory.** Word recall and word recognition was assessed with the same task material (Laukka et al., 2013). Participants were presented with 16 unrelated concrete nouns both orally and visually. Immediately following the presentation, they were asked to freely recall as many words from the learning list as they could within a two-minute time frame. The score for word recall was determined by the number of correctly recalled words. Following this, participants engaged in a self-paced word recognition task. They were presented with a mix of 16 target words and 16 distractors, and their task was to indicate whether they recognized each word. Performance in word recognition was assessed as the number of correctly recognized targets minus falsely recognized lures (hits - false alarms).

**Perceptual speed.** In the digit cancellation task (Zazzo, 1974), participants crossed out as many instances as possible of the number 4 (in rows of numbers) within 30 seconds. The number of correct identifications was taken as performance. In the pattern comparison task (Salthouse & Babcock, 1991), participants compared line-segment patterns to determine if a pair was "same" or "different." Performance was measured as the mean number of correct classifications across two pages, with 30 seconds allotted for each page.

**Semantic Memory.** Semantic memory was assessed using the Dureman-Salde battery's 30-item vocabulary test (SRB:1; Dureman, 1960; Nilsson et al., 1997). Participants identified synonyms for target words from sets of five words. The score was based on number of correct identifications within 7 minutes.

**Semantic Fluency: Letter Fluency.** Participants had 60 seconds to generate as many words as possible starting with the letters F or A, respectively. The number of unique correct words for each letter was recorded.

**Semantic fluency: Category Fluency.** Within a 60 s time frame, participants generated as many words as possible representing animals or professions. The number of unrepeated and correct words was registered for each category.

**Description of Control variables**

**Age** – Number of years since birth

**Gender -** Female vs. male

**Education** - Number of years of education

The first three covariates were chosen as the set of limited control variables for our models. These are the standard covariates within grandparenthood research (e.g., Henning et al., 2023)

**Work status –** Work status was recoded in working versus not currently working.

**Grip strength –** Grip strength was assessed with the Grippit (Nordenskiöld & Grimby, 1993). The participant squeezed a handle with maximum force, once with each hand, with best overall value being used in the analysis.

Work status and grip strength (or other measure of health) are also two common control variables reported within grandparenthood research. For work status, note however, that among the old-old adults, most of the participants were not working (98% in the old-old group, versus 59.34% of participants not working in the young-old group). The health measure, although most often added in previous research as a covariate, could also be the mechanism through which grandchild care might have an effect on cognition (Liao et al., 2021). Therefore, it was not included in the set of limited controls.

**General frequency of meeting with social network** was calculated by averaging the reported frequency of contact face-to-face and remote with the following members: Friends, neighbors, parents, siblings, other relatives (but not grandchildren or children). For each member, the response options were on a 6-point scale (where 1 = Daily, more than twice/week 2 = Weekly, more than twice/month; 3 = Monthly, more than 6 times/year; 4 = Quarterly, more than once/year; 5 = Less often; 6 = Never). The variable was added as a covariate to see if grandchild contact has any effects on cognition beyond the effects of general social contact. We reverse coded the items such that a high score would indicate high frequency of contact, and created an average over the responses given for each member (e.g., friends, parents).

**Time spent living alone** was calculated by the number of assessment waves grandparents reported living alone during the study period, that is without a partner or other members of one’s social network. Note that in the pre-registration we wrote years instead of waves – that was a mistake. We included this variable as factors such as partnership status have been linked to the importance allocated to the grandparenthood role (Mahne & Motel-Klingebiel), and living situation is also linked to cognitive functioning (Zahodne et al., 2019).

**Comparing Participants with Full versus Participants with At Least 1 Wave of Missing Data**

Compared to old-old adults with full completed waves, old-old adults with at least one wave missing have lower levels of cognition at first wave (54.12 for participants with all waves vs 49.04 for participants with at least 1 missing; *p* < .001), showed more cognitive decline (−1.47 vs −4.77; *p* < .001), and were older (80.13 vs 83.73; *p* < .001). However, they did not differ in grip strength (216.11 vs 202.59; *p* = .181).

Compared to young-old adult with all waves completed, those with at least one wave missing had lower cognitive functioning at first wave (51.31 vs 47.67; *p* < .001), showed more cognitive decline (−0.61 vs −2.37; *p* < .001), and were older (64.91 vs 67.30; *p* < .001). They again did not differ in grip strength (294.55 vs 284.13; *p* = .145).

**Adding Quadratic Change for Cognitive Performance in Latent Growth Curve Models**

We checked if cognitive performance is non-linear by adding a quadratic slope in the latent growth curve models (LGM models), in addition to the linear slope. The model with a quadratic slope for the young-old adults did not converge, thus we report only results for the models including only old-old adults.

We first run LGM models with a quadratic slope and no predictors. In accordance with the AIC and BIC, for old-old adults the model including also a quadratic slope would be preferred (see Table S1). Thus, we proceeded by predicting the quadratic slope by the same predictors as we did with the linear slope. However, only one effect was significant: Number of waves spent living alone negatively predicted the quadratic slope (ρ = −.060, *p* = .007). Number of waves spent living alone was a control variable in our study. Given this, and the uncertainty surrounding the effect of predictors on non-linear slopes, we do not follow with interpreting and discussing this finding. Nevertheless, our variables of interest (face-to-face and remote contact) did not predict the quadratic slope.

Table S1

*Comparison of LGM Models with and without quadratic slope*

|  | AIC | BIC |
| --- | --- | --- |
| **Old-Old Grandparents** |  |  |
| Linear model | 11416.383 | 11461.965 |
| Quadratic model | 11330.918 | 11394.733 |

**OSF Table S1**

*Model Fit Indices for Unconstrained and Constrained (Random) Intercept Cross-Lagged Panel Models*

|  | **Constrained Model** | | | | | **Unconstrained Model** | | | | |
| --- | --- | --- | --- | --- | --- | --- | --- | --- | --- | --- |
| **CLPM** | **CFI** | **RMSEA** | **SRMR** | **AIC** | **BIC** | **CFI** | **RMSEA** | **SRMR** | **AIC** | **BIC** |
| **Young-old** |  |  |  |  |  |  |  |  |  |  |
| Face-to-face | .982 | .072 | .018 | 41600.043 | 42070.331 | .983 | .111 | .016 | 41602.468 | 42102.775 |
| Remote | .984 | .066 | .016 | 41380.308 | 41850.596 | .987 | .094 | .012 | 41377.801 | 41878.108 |
|  |  |  |  |  |  |  |  |  |  |  |
| **Old-old** |  |  |  |  |  |  |  |  |  |  |
| Face-to-Face | .991 | .035 | .026 | 27161.113 | 27671.632 | .989 | .053 | .021 | 27176.749 | 27737.408 |
| Remote | .976 | .040 | .068 | 28026.593 | 28587.251 | .981 | .067 | .029 | 27113.468 | 27674.127 |
|  | **Constrained Model** | | | | | **Unconstrained Model** | | | | |
| **RI-CLPM** | **CFI** | **RMSEA** | **SRMR** | **AIC** | **BIC** | **CFI** | **RMSEA** | **SRMR** | **AIC** | **BIC** |
| **Young-old** |  |  |  |  |  |  |  |  |  |  |
| Face-to-face | 1.000 | .000 | .007 | 41638.250 | 42123.548 | 1.000 | .000 | .000 | 41644.393 | 42159.709 |
| Remote | 1.000 | .005 | .009 | 42157.252 | 42642.549 | 1.000 | .026 | .004 | 42163.767 | 42679.082 |
|  |  |  |  |  |  |  |  |  |  |  |
| **Old-old** |  |  |  |  |  |  |  |  |  |  |
| Face-to-Face | 1.000 | .000 | .022 | 27359.636 | 27883.828 | - | - | - | - | - |
| Remote | .997 | .021 | .028 | 27583.906 | 28108.099 | .999 | .015 | .017 | 27590.118 | 28164.451 |

*Note.* Face-to-face = Models for face-to-face contact, Remote = Models for remote contact, RMSEA = Root Mean Square Error of Approximation, CFI = Comparative Fit Index, SRMR = Standardized Root Mean Square Residual; AIC = Akaike information criterion, BIC = Bayesian information criterion. Unconstrained model for old-old adults did not converge.

**OSF Table S2**

*Model Fit Indices for Latent Growth Curve Models (No Predictors)*

| **LGM** | **CFI** | **RMSEA** | **SRMR** |
| --- | --- | --- | --- |
| **Young-old** |  |  |  |
| Cognition | .965 | .180 | .043 |
| Contact Face-to-face | 1.000 | .000 | .001 |
| Remote Contact | 1.000 | .000 | .007 |
| **Old-old** |  |  |  |
| Cognition | .939 | .112 | .066 |
| Contact Face-to-face | .994 | .020 | .083 |
| Remote Contact | .998 | .010 | .064 |

*Note.* Face-to-face = Models for face-to-face contact, Remote =

Models for remote contact, RMSEA = Root Mean Square Error of Approximation,

CFI = Comparative Fit Index, SRMR = Standardized Root Mean Square Residual.

**OSF Table S3**

*Model Fit Indices for Latent Growth Curve Models with Grandchild Contact as Predictor and All Covariates*

| **LGM** | **CFI** | **RMSEA** | **SRMR** |
| --- | --- | --- | --- |
| **Young-old** |  |  |  |
| Face-to-face | .963 | .096 | .016 |
| Remote | .964 | .096 | .016 |
|  |  |  |  |
| **Old-old** |  |  |  |
| Face-to-Face | .949 | .061 | .034 |
| Remote | .948 | .062 | .034 |

*Note.* Face-to-face = Models for face-to-face contact, Remote =

Models for remote contact, RMSEA = Root Mean Square Error of Approximation,

CFI = Comparative Fit Index, SRMR = Standardized Root Mean Square Residual.

**OSF Table S4**

*Model Fit Indices for Latent Growth Curve Models with Grandchild Contact as Predictor and Limited Covariates*

| **LGM** | **CFI** | **RMSEA** | **SRMR** |
| --- | --- | --- | --- |
| **Young-old** |  |  |  |
| Face-to-face | .963 | .128 | .024 |
| Remote | .964 | .127 | .025 |
|  |  |  |  |
| **Old-old** |  |  |  |
| Face-to-Face | .948 | .075 | .047 |
| Remote | .947 | .076 | .047 |

*Note.* Face-to-face = Models for face-to-face contact, Remote =

Models for remote contact, RMSEA = Root Mean Square Error of Approximation,

CFI = Comparative Fit Index, SRMR = Standardized Root Mean Square Residual.

**OSF Table S5**

*LGM Results: Covariates Associations with Cognitive Levels, and Cognitive Decline for Young-Old Adults*

| Model Young-Old |  | cov | r | *p* |
| --- | --- | --- | --- | --- |
| Level-level association for cognition and | Age | -.469 | -.074 | *.*030 |
|  | Sex (w) | 1.659 | .261 | < .001 |
|  | Education years | 2.688 | .424 | < .001 |
|  | Grip Strength | 1.124 | .177 | < .001 |
|  | Social network | .483 | .076 | .004 |
|  | Waves spent living alone | -.048 | -.008 | .785 |
|  | Work status (yes) | .679 | .107 | .001 |
|  |  |  |  |  |
|  |  | B | β | *p* |
| Predictors for slope cognition | Age | -.231 | -.249 | <.001 |
|  | Sex (w) | .079 | .085 | .247 |
|  | Education years | -.041 | -.044 | .300 |
|  | Grip Strength | -.028 | -.030 | .672 |
|  | Social network | .053 | .058 | .185 |
|  | Waves spent living alone | -.116 | -.125 | .004 |
|  | Work status (yes) | .080 | .086 | .115 |

*Note.* B *=* unstandardized regression weight; β = standardized regression weight. Age, sex, education, social contact, waves spent living alone, and work status were z-standardized, and thus the values represent the difference in T-scores based on the difference in one SD of the covariates; Sex (w), where 0 = men and 1 = women; Social network = Frequency of contact with members of social network, other than grandchildren; Waves spent living alone = Number of waves living alone; Work status (yes), where 0 = not working and 1 = working.

**OSF Table S6**

*LGM Results: Covariates Associations with Cognitive Levels, and Cognitive Decline for Old-Old Adults*

| Model Old-Old |  | cov | r | *p* |
| --- | --- | --- | --- | --- |
| Level-level association for cognition and | Age | -1.677 | -.251 | < .001 |
|  | Sex (w) | .637 | .095 | .105 |
|  | Education years | 2.760 | .413 | < .001 |
|  | Grip Strength | .792 | .120 | .058 |
|  | Social network | 1.116 | .167 | < .001 |
|  | Waves spent living alone | 1.114 | .167 | < .001 |
|  | Work status (yes) | .151 | .014 | .670 |
|  |  |  |  |  |
|  |  | B | β | *p* |
| Predictors for slope cognition | Age | -.393 | -.276 | .002 |
|  | Sex (w) | .173 | .122 | .313 |
|  | Education years | -.010 | -.007 | .925 |
|  | Grip Strength | .190 | .136 | .253 |
|  | Social network | -.120 | -.084 | .243 |
|  | Waves spent living alone | .133 | .093 | .219 |
|  | Work status (yes) | .079 | .055 | .371 |

*Note.* B *=* unstandardized regression weight; β = standardized regression weight. Age, sex, education, social contact, waves spent living alone, and work status were z-standardized, and thus the values represent the difference in T-scores based on the difference in one SD of the covariates; Sex (w), where 0 = men and 1 = women; Social network = Frequency of contact with members of social network, other than grandchildren; Waves spent living alone = Number of waves living alone; Work status (yes), where 0 = not working and 1 = working.

**OSF Figure S1**

**Model Results with Limited Covariates**

**
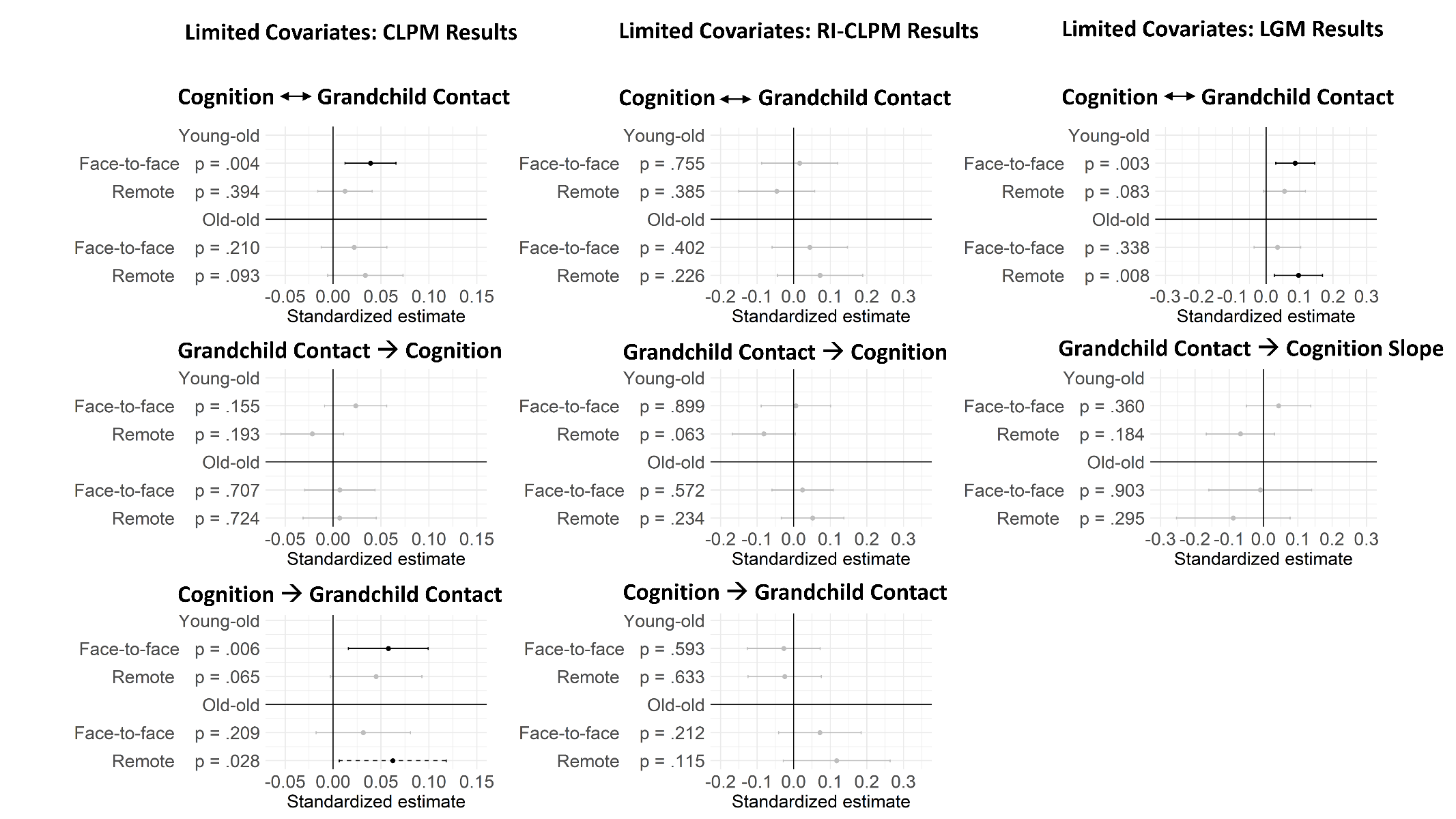
**

*Note*. Limited control variables included: age, gender, education; CLPM = Cross-lagged panel model with limited covariates; Grandchild Contact 🡨🡪 Cognition = Within time-points associations between grandchild contact and cognition; Grandchild Contact 🡪 Cognition = Effects from frequency of grandchild contact on subsequent cognition; Cognition 🡪 Grandchild Contact = Effect from cognition to subsequent grandchild contact; RI-CLPM = Random-intercept cross-lagged panel model with limited covariates. LGM: Latent Growth curve model; Grandchild contact 🡪 Cognition Slope = Grandchild contact predicting change in cognitive decline over time.

**References**

Dureman, I. (1960). SRB: 1. *Stockholm: Psykologiförlaget*.

Henning, G., Ehrlich, U., Gow, A. J., Kelle, N., & Muniz-Terrera, G. (2023). Longitudinal associations of volunteering, grandparenting, and family care with processing speed: A gender perspective on prosocial activity and cognitive aging in the second half of life. *Psychology and Aging*.

Laukka, E. J., Lövdén, M., Herlitz, A., Karlsson, S., Ferencz, B., Pantzar, A., ... & Bäckman, L. (2013). Genetic effects on old-age cognitive functioning: a population-based study. *Psychology and aging*, *28*(1), 262.

Laukka, E. J., Köhncke, Y., Papenberg, G., Fratiglioni, L., & Bäckman, L. (2020). Combined genetic influences on episodic memory decline in older adults without dementia. *Neuropsychology*, *34*(6), 654.

Liao, S., Qi, L., Xiong, J., Yan, J., & Wang, R. (2021). Intergenerational ties in context: association between caring for grandchildren and cognitive function in middle-aged and older Chinese. *International Journal of Environmental Research and Public Health*, *18*(1), 21.

Mahne, K., & Motel-Klingebiel, A. (2012). The importance of the grandparent role—A class specific phenomenon? Evidence from Germany. *Advances in life course research*, *17*(3), 145-155.

Nilsson, L. G., BÄCkman, L., Erngrund, K., Nyberg, L., Adolfsson, R., Bucht, G., ... & Winblad, B. (1997). The Betula prospective cohort study: Memory, health, and aging. *Aging, Neuropsychology, and Cognition*, *4*(1), 1-32.

Nordenskiöld, U. M., & Grimby, G. (1993). Grip force in patients with rheumatoid arthritis and fibromyalgia and in healthy subjects. A study with the Grippit instrument. *Scandinavian journal of rheumatology*, *22*(1), 14-19.

Salthouse, T. A., & Babcock, R. L. (1991). Decomposing adult age differences in working memory. *Developmental psychology*, *27*(5), 763.

Zahodne, L. B., Ajrouch, K. J., Sharifian, N., & Antonucci, T. C. (2019). Social relations and age-related change in memory. *Psychology and Aging*, *34*(6), 751

Zazzo, R. (1974). Test Des Deux Barrages. Actualités Pédagogiques EtPsychologiques (Vol. 7). Delachaux Et Nestlé.
